# *Die Fledermaus*: Regarding Optokinetic Contrast Sensitivity and Light-Adaptation, Chicks Are Mice with Wings

**DOI:** 10.1371/journal.pone.0075375

**Published:** 2013-09-30

**Authors:** Qing Shi, William K. Stell

**Affiliations:** 1 Neuroscience Graduate Program, University of Calgary Faculty of Medicine, Calgary, Alberta, Canada; 2 Department of Cell Biology and Anatomy, and Department of Surgery, and Hotchkiss Brain Institute and Alberta Children’s Hospital Research Institute; University of Calgary Faculty of Medicine, Calgary, Alberta, Canada; Oregon Health & Science University, United States of America

## Abstract

**Background:**

Through adaptation, animals can function visually under an extremely broad range of light intensities. Light adaptation starts in the retina, through shifts in photoreceptor sensitivity and kinetics plus modulation of visual processing in retinal circuits. Although considerable research has been conducted on retinal adaptation in nocturnal species with rod-dominated retinas, such as the mouse, little is known about how cone-dominated avian retinas adapt to changes in mean light intensity.

**Methodology/Principal Findings:**

We used the optokinetic response to characterize contrast sensitivity (CS) in the chick retina as a function of spatial frequency and temporal frequency at different mean light intensities. We found that: 1) daytime, cone-driven CS was tuned to spatial frequency; 2) nighttime, presumably rod-driven CS was tuned to *temporal frequency* and spatial frequency; 3) daytime, presumably cone-driven CS at threshold intensity was invariant with temporal and spatial frequency; and 4) daytime photopic CS was invariant with clock time.

**Conclusion/Significance:**

Light- and dark-adaptational changes in CS were investigated comprehensively for the first time in the cone-dominated retina of an avian, diurnal species. The chick retina, like the mouse retina, adapts by using a “day/night” or “cone/rod” switch in tuning preference during changes in lighting conditions. The chick optokinetic response is an attractive model for noninvasive, behavioral studies of adaptation in retinal circuitry in health and disease.

## Introduction

Vision functions over a vast range of light intensities, as much as 14 log_10_ units [1]. For species survival, it is advantageous to use as much as possible of the available intensity range for vision. Animals are able to do this – to see effectively over a range of light intensities, from weak starlight to brilliant sunshine – because visual sensitivity and gain can adjust automatically to ambient light intensity, thus optimizing visual function under widely varying conditions. This property of vision is called *adaptation*. In the retina, adaptation depends in part on the duality of photoreceptor systems – rod photoreceptors, which mediate vision in relatively low-intensity (*scotopic*) conditions, and cone photoreceptors, which mediate vision in relatively high-intensity (*photopic*) conditions, with the two functioning together in an intermediate (*mesopic*) range. However, retinal circuitry also changes functionally – from high sensitivity and low acuity at low intensity, to low sensitivity and high acuity at high intensity. This switching between rod- and cone-driven retinal circuits, and the adjusting of the sensitivity and gain of both photoreceptor responses and post-receptoral circuits, are the main factors that make useful vision possible over such a wide range of light intensities.

In the present study, we have investigated the effects of light- and dark-adaptation on visual processing in the chick retina, using the optokinetic response (OKR). In animals with laterally placed eyes, such as mice and chickens, the OKR is a simple, unlearned reflex turning of the head and neck (therefore also called the “optocollic” response) to follow the rotation of a global visual pattern in the horizontal plane. It is quite simple to determine the minimum contrast (threshold) at which the animal can follow a vertical stripe pattern (grating) of known spatial frequency, contrast and velocity, on a cylindrical surface rotating around it. Although the OKR is modulated by connections within the brain, especially from the vestibulocerebellum [2], its contrast sensitivity (CS), gain, and response to the direction and speed of image movement are determined largely by the function of a single class of directionally selective retinal ganglion cells (DS-RGC) [3]. In the horizontal OKR, these cells respond preferentially to object movement in the temporonasal direction, and when they are activated in one eye, they are silent in the other. Therefore, one can test alternately the retinal function in each eye independently, simply by reversing the direction of movement [4]. The recent introduction of a “virtual optomotor system” (OptoMotry®) – in which spatial frequency, contrast, velocity, and intensity of a computer-generated drifting grating can be changed instantly and continuously over a wide range – makes it possible to measure optokinetic CS as a function of these parameters, rapidly and easily [5]. As a result, this method has become standard for characterizing normal and experimentally altered retinal function in small animals – in particular, mouse [6]–[13], rat [4], [14], [15], Nile grass rat [16], and even zebrafish [17].

Using the OKR, Umino et al. [6] have found that CS of the light-adapted mouse is tuned to velocity, whereas CS of the dark-adapted mouse is tuned to temporal frequency (and both, to spatial frequency). That is, *the tuning preference of the mouse retina switches under different adaptational states*. In extremely dim-light conditions, rod-dependent signals in a typical mammalian retina are relayed indirectly: rods → rod bipolar cells (BCs) → AII amacrine cells → cone BCs → RGCs; in contrast, cone-dependent signals go through a more direct pathway: cones → cone BCs → RGCs. It is tempting to imagine that this switch of tuning preference between light- and dark-adaptation involves the unique rod-specific pathway of the mammalian retina – comprising rod bipolar cells and their AII-amacrine cell relay – under scotopic conditions, and its removal in favor of cone pathways under photopic conditions. Certainly, dark-adaptation brings dramatic changes in cell activity and cell-cell coupling in the rod-specific pathway [18], which should alter the way visual information (such as spatial contrast and detail) is processed in the inner-retinal circuits that control DS-RGCs. However, while much is known about the neural circuitry and function of DS-RGCs under light-adapted conditions [19], effectively nothing is known about how they change with dark-adaptation.

The chick is an attractive alternative to mice and other common laboratory mammals for studying such mechanisms. Chickens (as opposed to the ubiquitous laboratory mouse and rat models) have excellent cone-based vision from the time of hatching, and their retinal function as revealed by the OKR reaches a stable adult level by 5–7 days after hatching (P5–P7) [20]. These young chicks are small, docile, and very suitable for testing in OptoMotry. While most neurobiological studies of retinal mechanisms for adaptation have been carried out in animals having rod-dominated retinas, it is of biological interest also to know how retinal functions adapt in cone-dominated retinas. The chicken retina is cone-dominated, with cones in some strains comprising ∼86% of photoreceptors in the center and ∼70% in the periphery of the retina [21]. Furthermore, while powerful methods for manipulating gene expression (as in mice) are not yet available for birds, the large size of chick eyes makes it easy to manipulate retinal function – *one eye at a time, independently*– by the equally powerful method of delivering pharmacological agents preferentially to the retina by intravitreal injection [22], [23].

The spatial visual function of birds, assessed as CS, has been documented by electrophysiological [24] as well as behavioral methods [20], [25]. All of these studies have reported that the photopic CS in birds is tuned to spatial frequency (SF) – although the optimum SF and CS vary from study to study, possibly in part because of differences in (e.g.) methods (ERG vs. learned vs. innate behaviors), species being tested (e. g., barn owls vs. quails and pigeons), and ambient light intensity. In one study [25], *visual acuity* of the chicken was tested under five different luminances (from 0.06 to 57.35 cd/m^2^) using a classically conditioned, task-performance method, and was found to increase as light intensity was increased. However, how *spatial and temporal CS* change under different lighting conditions – that is, how spatiotemporal signaling adapts to maximize vision under different adaptational states – has not yet been studied in any avian species. Furthermore, although they differ from mice in having a strongly cone-dominated retina [21], the eyes of diurnal birds do have well documented rod function, which predominates at night; this has been detected by electroretinography, in the chicken [26] and the closely related Japanese quail [27]. Finally, vision in chicks is also of special interest because chicks have served for decades as the most-studied model of myopia [28], [29]. The chick is thus a perfect subject for further studies of these fundamental visual functions.

The observations reported here show that the functional strategy for optimizing visual function over a wide range of light intensities in the chick is remarkably similar to that in the mouse. Specifically, contrast sensitivity is high and tuned preferentially to fine detail (high SF) in the daytime, when environmental light is abundant, but switches to lower CS and tuning to coarser detail in the nighttime, when light is scarce. Some of these findings have been reported previously in abstract form [30].

## Materials and Methods

### Animals

White Leghorn cockerels (*Gallus gallus domesticus*) were purchased from Canadian hatcheries, delivered to us on post-hatching day 1 (P1), and tested on days P5–P13. For reasons of cost and availability, at various times we used chicks of 2 different strains: Lohmann (Pacific Pride Chicks, Ltd, Abbotsford, BC) and Bovan (Rochester Hatchery, Westlock, AB). Chicks were kept at 26°C on a 12∶12 hr light-dark cycle (light on at 06∶00 am) and had unlimited access to food and water.

### Ethics Statement

Animal use protocols were approved by the Health Sciences Animal Care Committee of the University of Calgary (Protocol #M10008), and complied with the CCAC Guide to the Care and Use of Experimental Animals as well as the ARVO Statement for the Use of Animals in Ophthalmic and Vision Research.

### Testing the Optokinetic Response (OKR)

#### Setup: OptoMotry®

The OKR was tested using a computer-operated virtual optomotor cylinder, OptoMotry® (Cerebral Mechanics, Lethbridge, AB, Canada), which generates horizontally drifting vertical gratings on the walls of a square enclosure formed by four 17-inch-diagonal flat-panel color LCD monitors (model 1703 FP; Dell, Phoenix, AZ). The grating waveform (sinusoidal in our experiments), horizontal drift velocity (V, deg/sec) and spatial frequency (SF, cycles/deg) – and thereby the temporal frequency (TF [ = SF x V], cycles/sec) – were controlled by the experimenter in software. Because of aliasing and other technical limitations, the upper limits of test parameters were SF = 1.0 cyc/deg and V = 50 deg/sec. Luminance was measured with a photometer (Minolta LS-110 Luminance Meter, operating in spot mode with a 1 degree acceptance angle), aimed horizontally in the place of an animal being tested. According to these measurements, in our experiments the maximum luminance of the light bars was 195 cd/m^2^, the minimum luminance of the dark bars was 2.91 cd/m^2^, and the mean luminance (of the entire grating) was 95 cd/m^2^, or 1.98 log cd/m^2^. This is about 4 log units below the luminance of bright sunlight [1]. However, it has been shown that at this luminance, chickens are strongly sensitive to long-wavelength light far beyond the rhodopsin spectral range [31], and therefore that this level of illumination is photopic for chickens. For testing at lower mean luminance, neutral-density (ND) filters (Lee Filters, Toronto, ON, Canada) in increments of ND = 0.5 or 0.9 were placed inside a transparent, cylindrical holder (inner diameter = 19.5 cm, outer diameter = 20.3 cm) between the monitors and the stand for the animal. The transparent cylinder alone had no measurable effect on CS function.

#### The OKR testing procedure

The OKR was tested under three light intensities: (i) in the day, at unattenuated mean luminance (I_mean = _1.98 log cd/m^2^), under which the OKR was driven by cones (defined as “photopic” condition); (ii) at night, at the lowest luminance under which an OKR could be elicited (defined as “scotopic condition”, or “nighttime threshold luminance”); and (iii) in the day, at the lowest luminance under which an OKR can be produced (defined as “daytime threshold luminance”). For scotopic testing, the chick was first dark-adapted in a dark room for at least 1.5 hours, then covered with an opaque black cloth and transferred quickly into the OptoMotry chamber. The scotopic OKR was viewed from above with an infrared-sensitive CMOS night-vision mini-camera (Model CM900, Clover Electronics, Cerritos, CA, USA), with infrared LEDs emitting outside the visible range (λ_max_ = 950 nm, no detectable emission at λ<800 nm), and the infrared LEDs were covered with 10 filters of ND = 0.9 each, to block completely any illumination of the chick by light within its visible range. The infrared camera was inserted through the lid of the OptoMotry chamber. Layers of black cloth were wrapped around all contacts between the filter cylinder and the testing chamber, to prevent illumination of the chick except by light passing through the ND filters; nevertheless, it is possible that a small amount of light leaked through, which could be detected by the chick’s retina but not by our instruments.

To determine the threshold luminance (for eliciting an OKR) under the above-described conditions (ii) and (iii), ND filters were inserted until the OKR to gratings of 100% contrast could not be elicited any more. Filters were then removed in 0.5 ND steps until the OKR reappeared, and the I_mean_ at which that occurred was defined as “threshold”.

The grating contrast was set initially at 100% and then lowered in a stepwise manner, that is, 100% → 50% → 25% → … (100/2^n^)%, holding SF and V constant, until the chick failed to respond. The chick was tested five times under the same stimulus conditions, and the response was accepted as reliable if the OKR was elicited in four of the five trials. Contrast was then reduced by one step, and the test procedure was repeated until the response rate failed to reach the 4-of-5 criterion. The lowest contrast at which the chick reliably produced an OKR was defined as threshold contrast, whose reciprocal (100/%contrast_thr_) is CS. We chose not to test threshold also with contrast ascending from near zero, or varying randomly, because we obtained results reliably and much more rapidly using the method just described, and because the determination of absolute threshold was not our objective. Contrast sensitivity was tested further, as just described, at a number of SFs and temporal frequencies (TFs), the latter of which was derived by calculating TF = SF x V.

#### Testing for circadian regulation – Effect of time of testing on daytime contrast sensitivity

Previous studies have shown that avian light sensitivity varies with clock time, that is, that light-sensitivity oscillates within the 24-hour cycle without an external cue such as light [26], [27]. Therefore, before spatial and temporal CS were characterized, CS was tested at different times of day to determine (i) whether there were circadian rhythms of CS in the chick, and (ii) whether we needed to test CS at a specific time of day. The CS of the OKR was tested under daytime photopic and threshold luminances, at SF = 0.5 cyc/deg and velocity (V) = 9 deg/sec, on the same chicks at 8∶00–8∶30 am, 12∶00–12∶30 pm, 4∶00–4∶30 pm, and 8∶00–8∶30 pm. To minimize disturbance of their regular light-dark cycle, chicks were fetched from the holding room 5–10 minutes before 6∶00 pm (their regular light-off time), left in the dark until testing, and put back in the dark immediately after the test run was completed.

### Data Analysis

CS was obtained by calculating the reciprocal of the threshold contrast (100/%contrast_thr_). The error bars in all graphs represent the standard deviations of the means. One-way ANOVA was performed to assess the significance of differences (p<0.05) among values at different independent variables (SF or TF) in each spatial and temporal CS function. Statistical analyses and graphing were performed using InStat™ version 3.1a and Prism™ version 5.0a, respectively, for Macintosh (GraphPad Software, Inc., LaJolla, CA, USA). The validity of parametric statistics was confirmed by testing for normal distribution of data, using InStat.

## Results

### Daytime, Cone-driven Contrast Sensitivity Function

**Figure 1:**
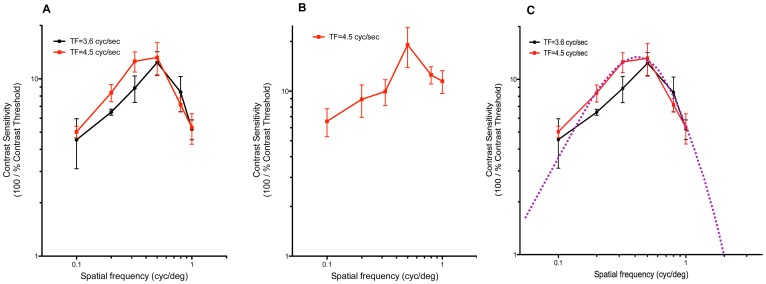
Examples of daytime, photopic spatial CS functions. (A) Bovan chicks (n = 6–8), (B) Lohmann chicks (n = 8). Contrast sensitivity peaks at about 0.5 cyc/deg. (C) Contrast sensitivity function for quail pERG (purple line; Ref. 24) scaled and fitted by eye to CS function of Bovan chicks (n = 6–8). Unattenuated mean luminance (I_mean = _1.98 log cd/m^2^) in all cases; mean ± SD. Peak CS is 13.2±2.8 in Bovan chicks (A, n = 8) and 19.1±5.2 in Lohmann chicks (B, n = 8), at ∼0.5 cyc/deg, and estimated SF_max_ (acuity) is ≥2 cyc/deg. TF, temporal frequency.

Photopic experiments were all performed in the daytime, from clock time 9 am to 3 pm, at 1.98 log cd/m^2^ (mean unattenuated luminance), contrast sensitivity (CS) was tested under a series of TFs (TF = 0.9, 1.8, 3.6, 4.5, 6.0 cyc/sec). In all chicks tested, regardless of strain (Bovan or Lohmann), the *spatial* CS function (CS *vs* SF) showed an inverted “U” shape (bandpass characteristic). Typical curves from each strain are shown in Figures 1A and 1B. This bandpass characteristic was seen at nearly all TFs tested (data not shown). Contrast sensitivity peaked at an intermediate SF, ca. 0.5 cyc/deg, where CS_max = _13.2±2.8 in Bovan chicks (n = 8) and 19.1±5.2 in Lohmann chicks (n = 8). Our findings suggest that the photopic, or cone-driven, CS of the chick was tuned to SF, and the optimum SF was about 0.5 cyc/deg. In Figure 1C, spatial acuity, the highest SF at which an OKR could be elicited under a specific light intensity, was estimated by curve-fitting, using the continuous function for CS vs SF of the quail’s pattern ERG [24]; this function was fitted by eye to the chick data, by adjusting position along the x-axis and adjusting x- and y-dimensions. CS in this range of SFs could not be tested using our setup, because of aliasing at SF >1.0 cyc/deg. While this method lacks quantitative rigor, it satisfies the need to visualize what the complete CS function might be and to estimate the spatial acuity of the photopic OKR. We estimated the acuity at maximum luminance to be about 2 cyc/deg in Bovan chicks (Fig. 1C), but data from Lohmann (Fig. 1B) and other chick strains (e.g., HyLine; data not shown) suggested that it could be as high as 6–8 cyc/deg.

**Figure 2:**
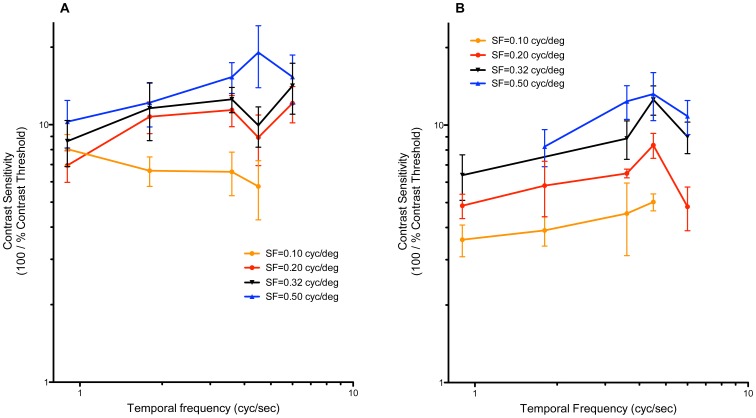
Daytime, photopic temporal CS functions. (A) Lohmann chicks (n = 6–8) and (B) Bovan chicks (n = 7–8), at unattenuated luminance (I_mean = _1.98 log cd/m^2^); mean ± SD. The CS functions of Lohmann chicks showed no statistically significant preference for any temporal frequency (A). In Bovan chicks, at SF = 0.2 and 0.32 cyc/deg, CS appeared to be bandpass, whereas at SF = 0.1 and 0.5 cyc/deg, they appeared to be more high-pass (at SF = 0.5 cyc/deg, difference in CS between the three highest TFs was insignificant, one-way ANOVA). SF, spatial frequency.

In contrast, photopic CS was not consistently tuned to *temporal* frequency (TF), in either Lohmann (Figure 2A) or Bovan (Figure 2B) chicks. For example, in Figure 2B, at SF = 0.1 and 0.5 cyc/deg, the CS function appears to be almost high-pass. At SF = 0.5 cyc/deg, difference in CS between the three highest TFs was insignificant (Figure 2B, between 1.8 and 3.6 cyc/s, p<0.05; between 1.8 and 4.5 cyc/s, p<0.01, one-way ANOVA). At SF = 0.2 and 0.32 cyc/deg, CS is more band-pass (p<0.05, one-way ANOVA).

### Chicks Function Visually Over A ≥6-Log cd/m^2^ Range of Light Intensity

Since previous ERG studies of the chicken [26] and quail [27] showed that rod functions predominated at threshold intensity in the nighttime, we determined the lowest intensity at which the OKR of the chick could be evoked at night. We found that the nighttime OKR could be elicited at −4.32 log cd/m^2^ (in Bovan chicks); this was 6.3 log cd/m^2^ lower than the maximum photopic luminance, 1.98 log cd/m^2^. It is of importance to note that 1.98 log cd/m^2^, the highest light intensity in our study, was far from that of the natural environment for diurnal birds such as chickens, being about 4 log units lower than that of full sunlight. Assuming that birds can see at that luminance, the real range of light intensities over which an OKR can be elicited would be >10 log units.

### Does Photopic Contrast Sensitivity Vary with Clock Time during the Day?

We wished to know whether CS of the chick varies in circadian fashion, as spectral sensitivity was found to do in previous studies [26], [27], and to determine whether our daytime experiments had to be performed in a short time period close to mid-day. For this, we determined the light-adapted, photopic CS of a single group of chicks at four times of day, that is, at 8∶00 am, 12∶00 pm, 4∶00 pm, and 8∶00 pm. The chicks were light-adapted for 1.5 hours before testing and then tested at *maximum* luminance. In contrast to light-sensitivity, the maximal photopic CS at SF = 0.5 cyc/deg was invariant with clock time (data not shown). To confirm further the lack of circadian rhythms in CS, we replicated the experiments under daytime *threshold* luminance at 8∶00 am, 12∶00 pm, and 4∶00 pm. Chicks being tested were dark-adapted for 1.5 hours prior to experiments. Contrast sensitivity was not tested at 8∶00 pm or later, because the OKR at this luminance in the nighttime would likely be driven by both rods and cones, if not by rods exclusively, and we did not want rod activity to influence the results. Again, no rhythms in CS were observed at different times (data not shown). Therefore, CS of the chick’s daytime OKR was not under circadian regulation, and we did not have to confine subsequent experiments to a short mid-day testing period.

### Nighttime Contrast Sensitivity Function

**Figure 3:**
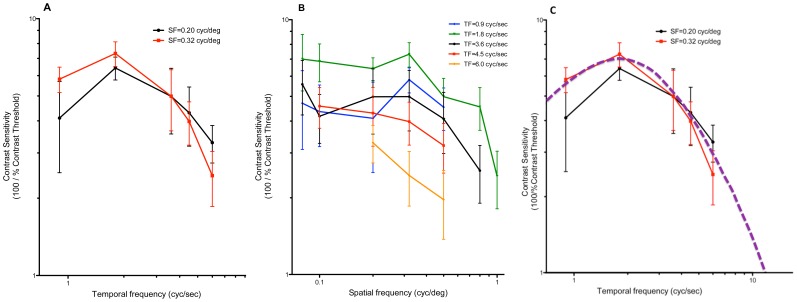
Nighttime, scotopic CS function of Lohmann chicks at minimal mean luminance (I_mean_ = −1.62 log cd/m^2^). (A) At the two spatial frequencies to which chicks were most sensitive, CS was clearly tuned to TF, with maximum CS = 7.32±0.804 at about 1.8 cyc/sec (n = 8–10). (B) In contrast, over a wide range of temporal frequencies, CS was poorly tuned to SF, with no significant dependence upon SF at any TF (n = 7–10). (C) Contrast sensitivity function for quail pERG (purple line; Ref. 24) scaled and fitted by eye to CS function of Lohmann chicks (n = 8–10). Estimated temporal acuity is 10–20 Hz.

The nighttime threshold luminance was determined by adding ND filters (see Methods) and observing the chicks’ responses in infrared (IR) light. In the nighttime (from 9∶00 pm to 12∶00 midnight), the threshold luminance for peak CS of the OKR was 3.6 log cd/m^2^ lower than that in daytime for the Lohmann chicks, and 6.3 log cd/m^2^ lower than that in daytime for the Bovan chicks; this indicated a large *increase in light-sensitivity* at night. Another difference from the daytime OKR was that at night, CS was tuned to TF but not to SF (Figures 3A and 3B). We present these data only for Lohmann chicks, even though their range of light-sensitivity was lower, because the nighttime OKR of the Lohmann chicks could be elicited over a wider range of SFs and TFs than that of the Bovan chicks. Contrast sensitivities at various SFs (tested at SF = 0.08, 0.1, 0.2, 0.32, 0.5, 0.8 cyc/deg) all peaked at a low-to-medium TF, 1.8 cyc/sec (Figure 3A), whereas CS did not vary consistently with SF at most SFs (Figure 3B). This clear bandpass characteristic was not seen in the daytime photopic CS function (Figures 2A and 2B). Additionally, the maximum CS was much lower in the nighttime (7.32±0.80) than in the daytime (19.1±5.16) (p = 0.0004, unpaired *t*-test). Moreover, the peak nighttime CS was found at a significantly lower SF (0.32 cyc/deg) than the peak daytime CS (0.5 cyc/deg), demonstrating a shift in spatial resolution (loss of CS at higher SFs) with adaptation from day to night. Finally, temporal acuity (a measure of highest temporal resolution), the highest TF at which an OKR could be elicited at a given light intensity, was estimated by curve-fitting (as with SF under photopic conditions; see above). While it could not be determined directly at the highest TFs, using our setup, in this way we estimated temporal acuity for the chick’s scotopic, rod-dominated OKR to be in the range of 10–20 Hz (Figure 3C).

### Daytime, Threshold-luminance Contrast Sensitivity

**Figure 4:**
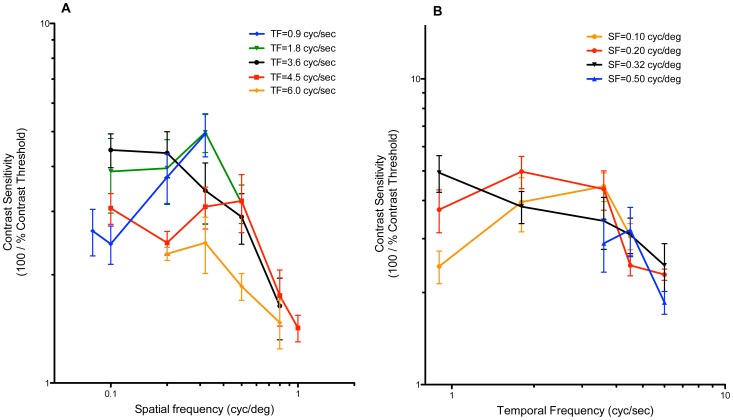
Daytime, scotopic CS of Bovan chicks (n = 6–7) at minimal mean luminance (I_mean_ = −2.12). No unique tuning characteristic could be discerned at this luminance, as low-pass, bandpass, and high-pass characteristics were seen in the spatial CS functions (A), and both bandpass and low-pass characteristics were seen in the temporal CS functions (B).

To determine whether rod-driven function was also not detectable in the daytime OKR, as suggested by the earlier ERG studies [26], [27], we determined the CS function at “threshold luminance” in the daytime. This threshold luminance (for the Bovan chicks) was −2.12 log cd/m^2^, that is, 4.1 log units lower than the unattenuated photopic luminance (1.98 log cd/m^2^). Interestingly, in these conditions CS did not vary consistently with either SF or TF, appearing to be tuned to SF at some TFs but not others (Figure 4A), and to TF at some SFs but not others (Figure 4B). At relatively high TFs – for example, 3.6, 4.5 or 6 cyc/sec – the shape of the CS-*vs*-SF curve suggested a low-pass characteristic (Figure 4A, p<0.0001, comparison between mean CSs at each SF tested, ordinary ANOVA); at TF = 1.8 cyc/sec, however, the curve showed a typical inverted U-shape or band-pass characteristic (P = 0.0015, Figure 4A), as did the photopic spatial CSF. At TF = 0.9 cyc/sec, CS increased monotonically as SF increased (Figure 4A). SF varies inversely with V; therefore, at TF = 0.9 cyc/sec, SF could be increased only by making the velocity too low for the chick to follow, and so CS could not be tested at still higher SFs.

These results led us to question whether rod-dependent functions were detectable only at night, because the daytime CS function at threshold luminance was clearly not like that which had been observed at either photopic or scotopic luminance. Either rod-dependent mechanisms had started to contribute to the OKR, or cone-dependent functions had been modified to a large degree, under this extremely low (yet higher-than-nighttime) threshold luminance. Further research will be needed to determine whether CS at daytime threshold luminance is driven by cones exclusively, or by a combination of cones and rods.

**Figure 5:**
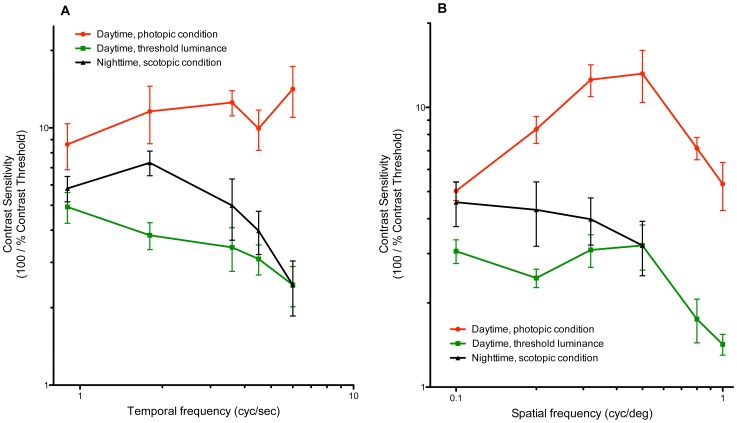
Contrast sensitivity functions under three conditions of adaptation and day-night cycle. (A) Temporal CS function at a specific SF (SF = 0.5 cyc/deg), under (i) daytime, photopic, (ii) daytime, threshold luminance, and (iii) nighttime, scotopic conditions. (B) Spatial CS functions at a specific TF (TF = 4.5 cyc/s), under the same three conditions as in (A).

To summarize, we found that both temporal and spatial contrast functions were modulated in different states of adaptation. For example, temporal CS exhibited a bandpass characteristic under scotopic conditions at night (Figure 5A) – but not in the daytime, whether light-adapted or dark-adapted. In contrast, spatial CS showed a bandpass characteristic only under photopic conditions in the daytime (Figure 5B) – but not under dark-adapted conditions, whether at night or in the daytime.

## Discussion

### Importance of Adaptation to Changes in Light Intensity

Through adaptation, animals are enabled to optimize their ability to survive and thrive in constantly fluctuating sensory environments. In the visual world, the most fundamental form of adaptation is to light intensity, which permits effective vision over a wide range of intensities. The retina uses enormous functional plasticity, dictated by ambient light levels, to extract the most useful information from visual images while discarding less useful information; the goal ultimately is to match visual function optimally to the needs- and opportunities! – imposed by the visual environment.

While the CS functions of various species have been explored in the light, only a few studies, e. g., psychophysics in human [33], [34] and macaque [34], and murine OKR [6], have investigated how it is affected by mean levels of illumination. Here we have used a rapid and reliable indicator of inner-retinal circuit function, the OKR, to test the CS function in the retina over a 6 log_10_ range of mean intensities. Interestingly, we have found that the scotopic OKR, tested at night, is tuned to TF, rather than to SF as when tested under photopic conditions during the day. A change in tuning preference at night indicates that the function of neural circuits in the retina has undergone major reorganization. In the retina of eutherian mammals, such as mice, rod signals are relayed in large part via a separate pathway that involves rod-only ON-bipolar and AII-amacrine cells [35]–[38]; this is bypassed when rods are inactive, under photopic conditions. Before undertaking the present studies, we assumed that switching to this rod-only pathway might account for the change in tuning of the OKR, from spatial to temporal frequency, which was observed in the mouse [6]. This led to the hypothesis that a comparable shift in tuning would *not* take place in the chick, because avian equivalents to the mammalian rod-only bipolar and AII-amacrine cells have not been identified so far [39], [40]. However, to our surprise the changes to spatiotemporal tuning of CS of the OKR with dark-adaptation in the chick were as profound as, and rather similar to, those in the mouse – and hence, the tongue-in-cheek title of this article.

### Different Strains of White Leghorns can be Very Different

We have noticed striking differences in the two strains of White Leghorn chicks used in the present study, such as absolute values of CS, behavior, etc. A major difference is that the Bovan chicks are much more sensitive to dim light at night than are the Lohmann chicks (a 2.7 log cd/m^2^ difference). One possible reason for this is that the Bovan chicks may have a higher rod:cone ratio than Lohmanns, since different rod:cone ratios have been reported previously in other strains [21], [41]. Different strains of chickens also were found to respond differently in myopia studies [42], indicating that processing of visual information in the retina may be expected to differ among strains, possibly as an inadvertent result of selection for other traits.

Inter-strain differences in pupil size also might contribute to differences in sensitivity However, since the difference in light sensitivity was found under extremely low light levels, this explanation would require the maximum pupillary area of the two strains to differ by ∼1000 times for the pupil-size theory to hold. Therefore, pupil size alone is very unlikely to explain such drastic difference.

### A Comparison with Results from Other Avian Studies

Previous studies from our lab have produced similar findings to these; in 6–12 day-old male White Leghorn (HyLine strain) chicks, optimal OKR CS was ∼10 (threshold 9.9%) at SF = 0.5 cyc/deg, with I_mean_ = 55 cd/m^2^ and V = 12 deg/sec [43], [44]. Schmid and Wildsoet [20] also have shown that the optokinetic CS of chicks ≤8 days old is tuned to SF, under conditions not very different from those in the present study. They reported that CS peaked at 1.2 cyc/deg; they likely missed the true maximum CS, which we have found to occur at about 0.5 cyc/deg, because their apparatus was not set up to test at any SF between 0.17 and 1.2 cyc/deg. In their study, the drift velocity was 6 deg/sec, the mean light intensity (I_max_) was 29 cd/m^2^, the contrast range was 4–78%, and the grating waveforms were sinusoidal from 0.12–1.7 cyc/deg. OptoMotry allowed us to test at an almost infinite number of contrasts, velocities and SFs, so that we could define the optimal parameters with greater precision than by any other method; however, our inability to test at SF >1 cyc/deg allowed us only to *estimate* spatial “acuity”, which could be defined with greater precision and tested at higher light intensities with a mechanical optokinetic cylinder such as that used by Schmid and Wildsoet, or a projected grating image such as that used by Schaeffel and colleagues [45]. Similarly, we estimate temporal “acuity”, the upper limit of responsiveness to temporal frequency, as 10–20 Hz (cyc/sec) – under nighttime scotopic conditions, the only ones in which tuning to TF was observed. This may seem rather low, since in another study [46] that used the flicker fusion frequency of the ERG and learned behavioral discriminations, the upper limit of flicker frequency perceived by Lohmann chickens was almost 120 Hz at high mean luminance (2740 cd/m^2^); however, at lower photopic luminance (0.7 cd/m^2^), the ERG flicker fusion frequency was 20 Hz [46]. Thus the estimated 10–20 Hz temporal acuity for the scotopic OKR observed in the present experiments (Figure 3C) seems plausible or even higher than might be expected, given that it was observed under presumably rod-dominant conditions, at approximately 1/4,000 the minimum luminance that was tested in the flicker-ERG experiments.

All things considered, despite the technical limitations and differences in apparatus used in these and other other studies, there is substantial agreement on fundamental properties: *at I_mean_ = 29–98 cd/m^2^ and V = 6–12 deg/sec, in 8–13-day-old male chicks of several White Leghorn strains, maximum contrast sensitivity is ca. 10–13, at SF = 0.5 cyc/deg, and spatial acuity is about 2–8 cyc/deg.*

Contrast sensitivity has been studied in several other species of birds, by a variety of methods. Ghim [47] and Ghim and Hodos [24], using threshold of the pattern ERG (pERG), reported the photopic CS of six species of birds; in the species most closely-related to chicken, Japanese quail, the peak CS was 9.85 at 1.05 cyc/deg. The difference in CS functions between their study and ours could not be explained simply by the difference in light intensities, since the mean luminances were almost the same (94 vs 98 cd/m^2^). A number of factors might have contributed to these differences: (1) The pERG stimuli were presented at sequentially ascending contrasts, sufficiently briefly (each one for 17 reversals at 7.5 Hz) that local contrast adaptation might have not been complete [48]; whereas our stimuli were presented at contrasts descending stepwise from 100%, and each contrast level was presented at least 5 times for 5 seconds, thereby creating a steady state of contrast adaptation and likely somewhat reduced CS. (2) The OKR is driven by retinal ganglion cells (ON DS-RGCs) that have rather large receptive fields and are not tuned optimally to fine detail, whereas the pERG represents the mixed responses of all retinal elements that are excited by pattern-reversal [49]. Since CS is determined by the relative strengths of center and surround processes, any elements that have a smaller receptive-field centre than those in the direction-selective pathway would have caused a shift of peak SF to the right, as seen in these pERG studies. Learned visual tasks require processing in higher visual centers that are more concerned with spatial discrimination *per se*. Thus Jarvis et al. [50], using a learned contrast-discrimination task (involving higher-level visual processing), found that the maximum CS of year-old chickens was slightly higher than 10– at highest luminance (16 cd/m^2^) and SF = 1 cyc/deg – and that when luminance was reduced to 0.1 cd/m^2^, peak CS declined to about 3–4 at 0.7 cyc/deg. Similarly, in mice, spatial acuity using the OKR [5] is considerably lower than that using a learned discrimination task [51].

### Comparison of CS in the Chick with CS in the Mouse

In vision research, the OKR (sometimes called optokinetic nystagmus, or optomotor response) is used widely as a measure of visual function in studies of disease and development [7], [8], [53]–[57]. It has also been employed as an indicator of drug effects in retinal pharmacology [58]–[62].

**Figure 6:**
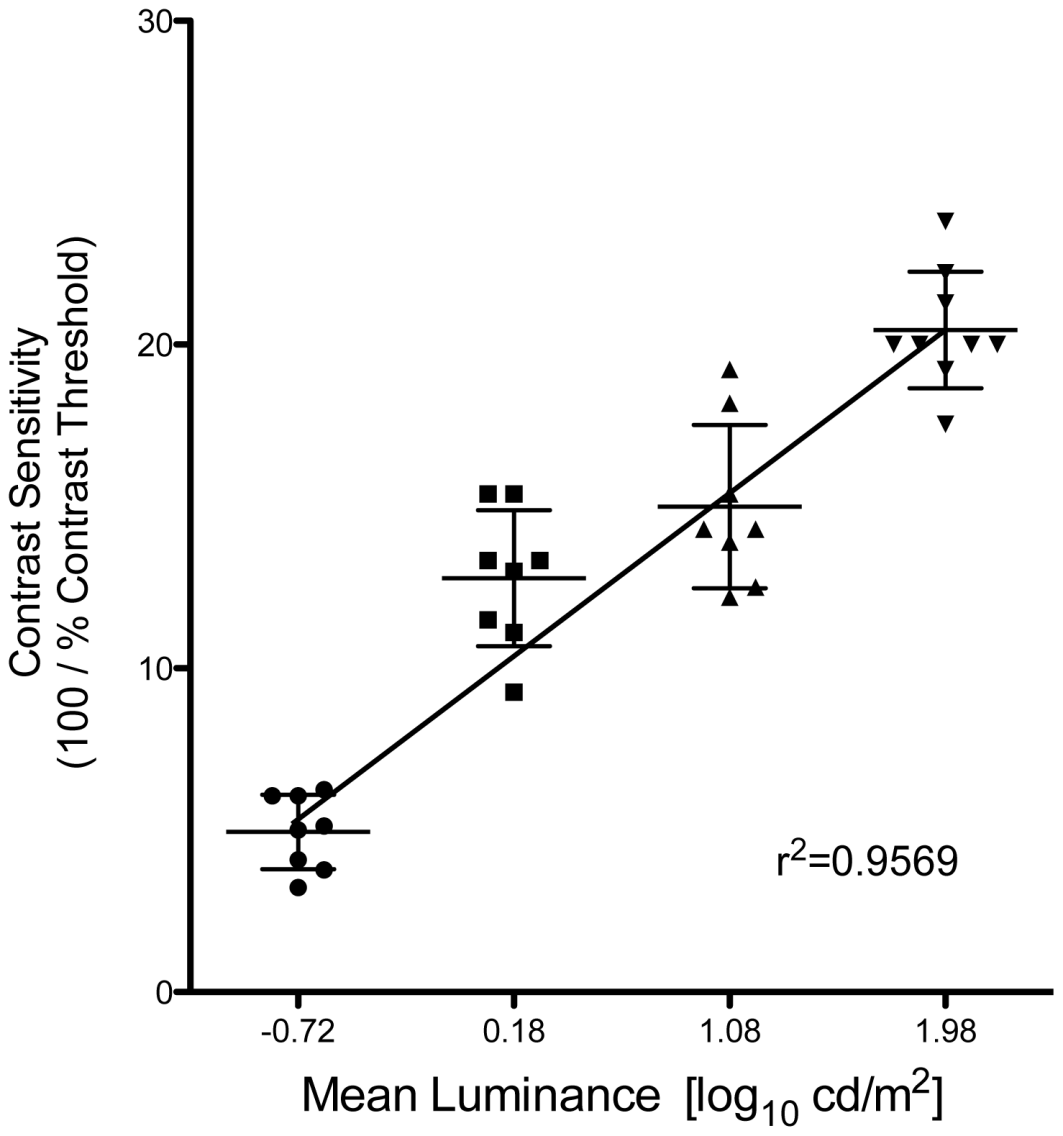
Photopic OKR vs mean luminance: In light- adapted chicks, with contrast sensitivity adaptated to steady state at test luminance for 30–60 min, CS declined linearly with log_10_ mean luminance (Weber’s Law). The critical threshold luminances, below which the photopic OKR was undetectable (in light-adapted chicks) and above which a scotopic OKR was elicited (in dark-adapted chicks), were statistically indistinguishable (P = 0.55) at −1.86 log_10_ cd/m^2^. Thus, rods and rod circuitry made no detectable contribution to even the scotopic OKR, under these daytime conditions.

However, only in the mouse has the OKR been used to characterize CS functions comprehensively under a wide range of environmental and visual stimulus conditions. The properties of CS in the chick, shown in the present report, are shared to a large extent with those of the mouse, measured by the OKR with a setup identical to ours [6]; that is, its photopic CS is tuned to SF of the visual stimulus, whereas its scotopic CS is tuned to TF. However, significant differences are also seen. For example, the peak CS under photopic conditions in the mouse is found near SF = 0.1 cyc/deg, which is much lower than that in the chick; this is likely due to the small fraction (≤5%) and sparse distribution of cones in the mouse retina, the correspondingly larger absolute diameter of receptive fields in the mouse retina, and the difference in optical magnification factors due to differences in eye size (ca. 150 µm/deg in chick: [62], [63]; 30 µm/deg in mouse: [64]). Additionally, in the study by Umino et al. [6], CS did not change much from the highest luminance (1.8 log cd/m^2^) to the second highest luminance tested (−2.7 log cd/m^2^), but changed substantially when luminance was reduced further from −2.7 to −4.5 log cd/m^2^. In contrast to this, in chicks we observed that CS at 0.5 cyc/deg decreased as a linear function of log I (Fig. 6), and when intensity was reduced by 5 log cd/m^2^, the CS function ceased being similar to that at maximum luminance. Moreover, the scotopic CS of the mouse was reported to vary as a distinct low-pass function of SF, which we did not observe in chicks. These differences may stem from differences in the functional organization of the retina in the two species – specifically, the existence of a dedicated rod pathway in mouse (mentioned earlier), which has not yet been discerned in avian retinas. Moreover, rod-dependent function (ERG) in quail and chick retinas has been reported to be observable only during the night [27], [26], suggesting that a circadian/retinal clock-dependent mechanism selectively suppresses rod function in the daytime in these diurnal species. In contrast, although evidence has been reported for circadian rhythms in the retinas of mice [65], [66] and several other vertebrates [67], [68], rod-dependent functioning in the mouse retina can be evoked in the daytime simply by adaptation to lower light intensity [6].

Finally, we note that the linear increase in CS with log I, up to the maximum intensity at which we could test, suggests that CS might continue to increase substantially with further increases in intensity. Since the absolute nighttime scotopic threshold I of the chick OKR is several log units higher than that of the mouse, it is logical to suppose that CS in birds is so remarkably low as emphasized by Ghim & Hodos [24] simply because the entire operating range of cone-dominated, diurnal avian vision is shifted several log units towards higher intensities compared to that of rod-dominated, nocturnal mammals such as the mouse. We predict, therefore, that testing of the chick’s CS at mean luminances on the order of 1,000–100,000 cd/m^2^ would reveal substantially greater maximum contrast sensitivities, comparable to those of many mammals.

### Is Nighttime Contrast Sensitivity Driven by Rods in the Chick?

Since it is practically impossible to determine whether the sustained scotopic luminance in the present study corresponds to the scotopic range for the flash ERG [27], because the units of light measurement in the two studies are not readily interconvertible, we could not definitely prove that the nighttime, threshold OKR was driven by rods. Moreover, the absolute scotopic range varies between different strains of a single species (see, e.g., our data for Bovan and Lohmann chicks).

However, such a correlation can be made between the mouse OKR and ERG studies. After converting light intensity to “retinal illuminance”, the scotopic range for the mouse OKR [6] correlates with that of the scotopic threshold response (STR) in an ERG study using the same strain of mice [69]. The STR, originating proximal to where the ERG b-wave is generated [70]–[72], appears at much weaker flash intensity than does the b-wave. Therefore, the rod-driven OKR of the mouse is produced *at light intensities below the b-wave threshold*. The findings from the ERG study of quail [27] were obtained under different light conditions, including those higher than required for the negative STR (see their Figures 2 and 5) – such as at b-wave levels. If the chick scotopic OKR was evoked at a light level equivalent to that which evokes the STR, as seen in the mouse [6]; [69], then our “scotopic” light intensity was not higher than the equivalent flash intensities in the chick and quail ERG studies; therefore, the nighttime threshold OKR is very likely to be driven by rods in the chick. To put this conclusion in context, one needs to bear in mind that the AII-amacrine cells in mammalian retinas are strongly coupled under very low light intensities at which rods function [18]. The high degree of spatial summation resulting from this coupling is thought to increase sensitivity and reduce noise at low light-levels, thus accounting for the low intensity threshold of the STR in the mammalian retina; but again, a comparable pathway or mechanism for increasing scotopic sensitivity has not yet been identified, and may not exist, in diurnal avian retinas.

### Contrast Sensitivity of Chick OKR does not Vary with Clock Hour during Daytime

It has been shown that photoreceptor responses, post-receptoral responses, and spectral sensitivity of Japanese quail varied with time of day [27] – even when they were being dark-adapted over days, indicating an endogenous circadian clock. This led us to wonder whether similar behavior could be seen in OKR contrast sensitivity in chicks. In fact, since CS is a measure of the ability to detect details or patterns, rather than mere light sensitivity, we had assumed that CS would be high in the daytime and low in the nighttime – with the peak at noon and the trough at midnight. This hypothesis was teleological or intuitive: in the daytime, when light is abundant, the retina needs to be less sensitive to light but can be more sensitive to small changes over space, time, and wavelength, facilitating more precise visual tasks such as spatial and temporal contrast and hue-discrimination; whereas at night, when photons are scarce, the most primitive function of the retina – simply to detect light with the highest sensitivity possible – outweighs all other demands, at the expense of those more photon-demanding photopic functions. However, we observed no variation of CS under constant lighting conditions during the daytime. This was not unexpected, as we tested throughout the daytime light phase at maximum luminance; and since light can override circadian rhythms (Figure 9 of [27]; Figures 1 and 3 of [67]), testing in the light-adapted, photopic state might have masked any regulation of optokinetic CS by an endogenous rhythm. To address this question, it will be necessary to keep the animals in constant darkness.

### Significance for Retinal Control of Ocular Growth and Myopia

It has been established that light controls eye growth through retinal signaling, and that only a few hours’ daily exposure to intense light, or to visual scenes rich in spatial detail (high SFs), prevents the induction of myopia [for data and review, see 73]. The primary mechanisms for the visual prevention of myopia reside in the retina, where a major role is played by amacrine cells [32], [74]. The effects of dark-adaptation on functional organization of the ON-center DS-RGCs appear not to have been studied [19], and how the avian retina adjusts physiologically during light- and dark-adaptation remains almost unknown, except for the ERG studies in chick [26] and quail [27]. This is a significant gap in knowledge, because of the major role of the chick as a model for human myopia plus the well-known importance of lighting, contrast and spatiotemporal processing in the cause and prevention of myopia [29]. Therefore, the present study adds further understanding of how light-adaptation alters retinal circuit functions, and it may direct our thinking into new areas of knowledge that are critical for preventing and treating myopia in the future.

## Conclusions

In the present study we used a rapid, noninvasive behavioral measure of visual function – the optokinetic response – to characterize retinal contrast sensitivity, under various light intensities and at different times of day and night. We found that the chick retina, like the mouse retina, showed a “day→night” or “cone→rod” switch in tuning preference, when adapting to the change from light to dark. This kind of change helps to optimize retinal functions under different lighting conditions. Finally, our study showed that all retinas, although different from species to species, might use simple and very similar mechanisms for light/dark adaptation. A better understanding of these conserved mechanisms awaits further exploration.
